# [Corrigendum] Influence of doxorubicin on apoptosis and oxidative stress in breast cancer cell lines

**DOI:** 10.3892/ijo.2026.5923

**Published:** 2026-07-20

**Authors:** Nesstor Pilco-Ferreto, Gloria M. Calaf

Int J Oncol 49: 753-762, 2016; DOI: 10.3892/ijo.2016.3558

Subsequently to the publication of the above article, an interested reader drew to the authors' attention that, concerning the three breast cancer cell lines investigated in their study, it was surprising that they should have detected caspase-3 expression in the MCF-7 cell line, as reported for example in Fig. 6, since it has been shown that MCF-7 cells do not express caspase-3 (see the paper by Reiner U. Jänicke and colleagues entitled 'MCF-7 breast carcinoma cells do not express caspase-3'. Breast Cancer Res Treat vol. 117.1 (2009); p. 219-221).

In their response, the authors acknowledge that the prevailing consensus in the literature, established by the seminal work of Jänicke *et al* in an article they published in 1998 in *Journal of Biological Chemistry* holds that MCF-7 cells harbor a 47-bp deletion in exon 3 of the CASP3 gene, resulting in a frameshift mutation and a premature stop codon, with consequent absence of detectable functional protein. The authors' own article acknowledged this controversy by citing conflicting reports (references 13 and 39 compared with references 41-43); however, they also recognize in retrospect that this should have prompted a more circumspect interpretation of their own data. Following a critical re-evaluation of their methodology, the authors wish to draw attention to the following limitations of the present work:

1. The band detected in MCF-7 cells using antibody H-277 cannot be unambiguously attributed to caspase-3, and cross-reactivity with co-expressed proteins of similar molecular weight, most notably caspase-7, cannot be excluded.
2. The DDRT-PCR amplicon obtained from MCF-7 cells with the reported primers may correspond to the aberrant CASP3 transcript arising from the exon 3 deletion, and does not necessarily represent a translation-competent mRNA. Sequencing of this amplicon was not performed, and would be required to resolve this question.
3. The conclusions of the article on caspase-3 expression in MCF-7 cells should therefore be read with these methodological caveats in mind. Importantly, these limitations do not affect the principal findings of the study concerning MDA-MB-231 and MCF-10F cell lines, for which the experimental evidence remains methodologically sound.

The authors are grateful to the Editorial Board of *International Journal of Oncology* for the rigor of the review process, and also for the opportunity to address these concerns transparently. They also thank the reader of the article for drawing these concerns to their attention.

